# Toward a better multi-model ensemble prediction of East Asian and Australasian precipitation during non-mature ENSO seasons

**DOI:** 10.1038/s41598-020-77482-4

**Published:** 2020-11-20

**Authors:** Soo-Jin Sohn, WonMoo Kim

**Affiliations:** Prediction Research Department, Climate Services and Research Division, APEC Climate Center (APCC), 12 Centum 7-ro, Haeundae-gu, Busan, 48058 Republic of Korea

**Keywords:** Climate sciences, Atmospheric science

## Abstract

An effective and reliable way for better predicting the seasonal Australasian and East Asian precipitation variability in a multi-model ensemble (MME) prediction system is newly designed, in relation to the performance of predicting El Niño-Southern Oscillation (ENSO) and its impact. While ENSO is a major predictability source of global and regional precipitation variation, the prediction skill of precipitation is not solely due to typical ENSO alone, of which variability and predictability exhibit strong seasonality. The first mode of ENSO variability has large variance with high prediction skill for boreal winter and small variance with low skill for spring and summer, while the second mode shows the opposite phase. The regional prediction skills for Australasian and East Asian precipitation also show such seasonal dependence, with low skill and large spread of individual models’ skills during the boreal spring to summer and high skill and small spread during winter. Using the individual models’ reproducibility of the association between ENSO and regional precipitation, the prediction skills of the MME with selected models can improve at regional levels, compared to those for all-inclusive MME, during boreal spring to summer. While typical ENSO as a predictability source may still dominate during boreal winter, consideration of complex ENSO structure and its diverse impact can lead to a better prediction of regional precipitation variability during non-mature phase of ENSO seasons.

## Introduction

The Asian monsoon is closely linked to the Australian counterpart, and the winter monsoon of one hemisphere feeds the summer monsoon of the other^1^. The fundamental natural driver of all monsoon systems is the differential solar heating during the spring season that helps establish land-sea thermal contrast and consequently trigger low-level moist convection. Another dominant interannual driver of Asian and Australian monsoon is El Niño-Southern Oscillation (ENSO)^2,3^. The ENSO bilaterally interacts with Asian-Australian monsoon system. It affects East Asian climate through the Pacific-East Asian teleconnection pattern^4^, the interannual variability of winter monsoon flow along the coastal East Asia feeds back to ENSO, and ENSO regulates the following summer monsoon in turn^5^. The Asian-Australian monsoon also plays a role in determining the onset time of ENSO^6^. However, Asian-Australian monsoon as well as ENSO-monsoon interaction is modulated in longer timescales, e.g., the winter climate over East Asia depends on the phase of the Pacific Decadal Oscillation (PDO)^7^ and the ENSO impact has become more diverse under climate change. There has been a decadal change in the East Asia-western North Pacific summer monsoon relationship from an ENSO-related oscillation in 1979–1993 to a monsoon-dominated oscillation in 1994–2004^8^. Therefore, understanding monsoon precipitation and improving the prediction accuracy still remain as a major scientific challenge.

It has been recognized that a dynamical seasonal prediction (DSP) based on general circulation models (GCMs) and their multi-model ensemble (MME) is a powerful tool for seasonal climate prediction. The main predictability in general originates from the ENSO and its well established climate impact^9,10^. However, the prediction of ENSO and its impacts are fundamentally challenged by the annual cycle of persistence^11,12^, and the tropics-midlatitude teleconnections sensitively depend on the location of maximum SST anomalies in the equatorial Pacific Ocean^13^. Studies show that the ENSO has complex features in space and time^14^, such that ENSO can be viewed as a superposition of at least two EOF modes of tropical Pacific SST anomalies. However, there have been relatively few diagnostic studies on the predictability of the complex ENSO features, as well as its impact over the East Asian and Australasian precipitation in DSP and MME. The MME, being a valuable and useful approach for cancelling model biases and offering better predictability^15^, generally performs better than a single individual model. However, the MME prediction skills for some seasons and regions are still relatively limited^16,17^. Here we particularly focus on the reproducibility of the complex ENSO structure and its impact on precipitation over East Asian and Australasian regions in coupled models and show how to utilize the information toward a better prediction of regional precipitation during non-mature phase of ENSO seasons.

### Implication of ENSO in regional prediction skill

**Figure 1:**
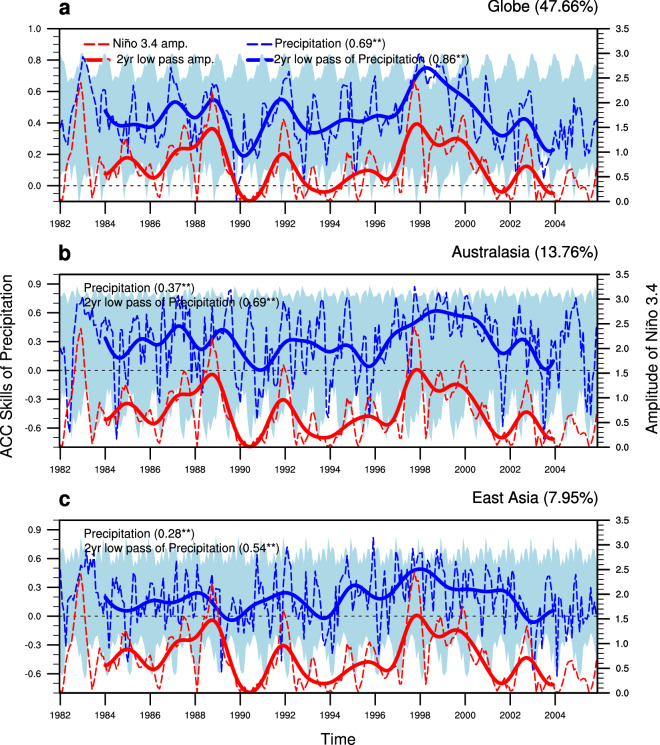
Time series of the anomaly pattern correlation coefficient (ACC) skill of (**a**) the one-month lead MME mean global (90°S-90°N, 0°-360°E), (**b**) Australasian (45°-10°S, 110°-180°E), and (**c**) East Asian (20°-60°N, 90°-150°E) precipitation prediction (blue dashed line for left y-axis) and the observed monthly three-month mean Niño 3.4 amplitude (red dashed line for right y-axis), within the period of 1982 to 2005. The area filled in light blue indicates the ACC skill distribution of cross-validated hindcasts associated with the targeted season and year of prediction. Solid lines show the 2-year low pass filtered data of the former two time series. Temporal correlation coefficients (TCCs) between the observed Niño 3.4 amplitudes and MME simulated precipitation skills are given in parentheses following the legends (with values exceeding the 1% significant level marked by double asterisks). The percentage variance of MME simulated precipitation skills explained by the observed Niño 3.4 amplitudes together are provided in parentheses following the labelled targeted domains of upper right of each panel (units: %). The figure was generated by NCAR Command Language (NCL)^28^.

Since ENSO is the primary source of atmospheric climate variability, the implication of ENSO in MME precipitation prediction skills has been first evaluated. Figure 1 shows the temporal fluctuation of MME precipitation skill based on the anomaly pattern correlation coefficient (ACC) between observations and the APEC Climate Center (APCC) MME 1-month-lead precipitation prediction in global, Australasian, and East Asian regions, based on all seasons of 24 years. The cross-validated ACC skills are calculated from the precipitation anomalies of the individual model predictions and observations for the targeted season and year (see Data and evaluation methods). The targeted MME ACC skills during 1982–2005 are within the range of cross-validated hindcast skills. As the predictive skills show a clear annual cycle, they fluctuate with region and season. It is clearly demonstrated that the regional MME prediction skills are relatively limited compared to the global one. The time-averaged global ACC value of MME 1-month-lead precipitation forecast is 0.44, which is significantly higher than the corresponding time-averaged values for Australasia (0.30) and East Asia (0.18).

However, the MME precipitation prediction skills for these regions are all significantly (at 99% confidence interval) correlated with the variation of the amplitude of observed Niño 3.4 index, albeit different degree of association, with correlation values of 0.69, 0.37, and 0.28 for the globe, Australasia, and East Asia, respectively. High skills are observed during the mature ENSO phases of 1982/1983, 1987/1988, and 1997/1998 when El Niño was strong. Removing the biennial signals from the precipitation prediction skills and the magnitude of Niño 3.4 SST index shows even higher correlation values of 0.86, 0.69, and 0.54. The respective degrees of association between the precipitation prediction skills and the strength of ENSO for the three regions are fairly explained by the corresponding fractional variance explained by ENSO. A typical ENSO approximately explains 48%, 14%, and 8% of the variance of precipitation prediction skills over the globe, Australasia, and East Asia. However, the discrepancy in the regions also confirms that although the predictability source of regional precipitation prediction is attributed to the variability of ENSO, it is not solely due to typical ENSO alone.

### Seasonality and predictability of ENSO

**Figure 2:**
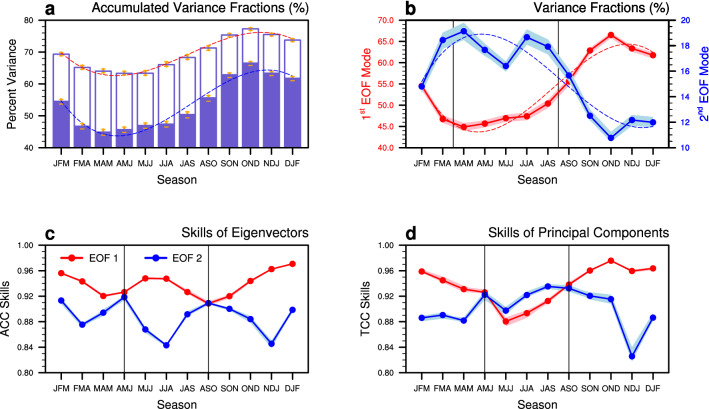
(**a**) Accumulated variance fractions explained by the first two leading modes of the observed ENSO as function of seasons (units: %). The range in orange indicates the variance fractional distribution of cross-validated anomalies associated with the targeted season and year. Slate blue and white parts of each bar denote the first and second modes, respectively. Red and blue dashed lines show the least squared polynomial fit to the whole accumulated and first leading variance, respectively. (**b**) Variance fractions of the 1st (red circled line for left y-axis) and 2nd EOF modes (blue circled line for right y-axis) for the observed ENSO. Red and blue dashed lines show the least squared polynomial fit to the former two time series. Skills of the one-month lead MME mean ENSO for (**c**) eigenvectors and (**d**) principal components (PCs). The forecast skills of the former two EOF components are defined by ACC for eigenvectors and TCC for PCs, respectively, between values from observations and MME simulation. In (**b**, **c**, **d**), the areas filled in pink and light blue indicate the distribution of cross-validated anomalies associated with the targeted season and year, and the vertical solid lines present the references for comparison. The figure was generated by NCL.

To consider the temporal and spatial structures of ENSO, we define ENSO as a superposition of the first two leading modes of the tropical SST anomalies (SSTAs) EOFs for each calendar season from January–February-March (JFM) to December-January–February (DJF) (see Data and evaluation methods), which may differ from the conventional definition. The first leading mode is typified by the warming in the eastern and central tropical Pacific, but the second mode is related to its zonal shift. They account for about 63–77% of the total variance, with large variance for boreal winter and small variance for spring and summer (Fig. 2a). The cosine-like variation, mainly due to the first leading principal component (PC), represents the seasonal phase locking of ENSO. It means that the anomalous warming/cooling in the eastern and central equatorial Pacific is the strongest during boreal winter and weakest during boreal spring. On the other hand, the second leading mode shows the opposite phase with smaller variance for boreal winter and larger variance for spring and summer (Fig. 2b). It implies that the relatively weak SSTAs persist in the central equatorial Pacific, but the weak signals are more likely to zonally shift during boreal spring and summer, which results in the difficulty in predicting the ENSO evolution during boreal spring^18^.

The predictability associated with the seasonal presence of temporal and spatial ENSO structures is also assessed (Fig. 2c,d). The MME simulation at 1-month lead in general well predicts the spatial–temporal features of the first two leading modes, but exhibits modally and seasonally different prediction skills. The MME shows the high fidelity in simulating the spatial structure of the first mode compared to the second mode, throughout the year. It indicates that the models can well predict the SST warming and cooling in the eastern and central tropical Pacific, rather than the zonal shift of SSTA fluctuation. The temporal prediction skill of the first mode also has seasonality and shows spring predictability barrier, *i.e.*, low predictive skill during spring and summer compared to the high predictive skill during boreal winter. Surprisingly, however, during boreal spring and summer the prediction of the second mode shows higher predictability than other seasons. It coincides with the inversion between annual cycles of the first two leading modes’ variance.

### Seasonal dependence of precipitation prediction skills

**Figure 3:**
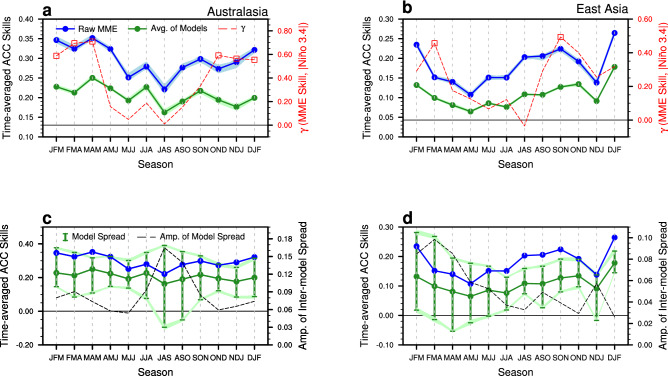
Time-averaged ACC skills (for left y-axis) of the model-simulated precipitation prediction for the (**a**, **c**) Australasia and (**b**, **d**) East Asia as a function of targeted forecast time. Solid blue circled and green circled lines of each panel denote the MME mean simulation skill and the average value of individual models’ skills, respectively. In (**a**, **b**), the areas filled in light blue and light green indicates the ACC skill distribution of cross-validated hindcasts associated with the targeted season and year of prediction. The red dashed line (for right y-axis) of (**a**, **b**) shows the TCC (γ) between the observed Niño 3.4 index amplitude and the ACC skill of MME mean simulations (with those exceeding the 5% significance level marked by the squares) for each season. The green bar (for left y-axis) and black dashed line (for right y-axis) of (**c**, **d**) show the spread of individual models’ skills and its amplitude, respectively. The figure was generated by NCL.

To what extent can the MME seasonal climate prediction capture the precipitation variability over the Australasian and East Asian region? In this section, the current status of precipitation forecast over Australasia and East Asia is examined from the seven coupled models and their MME. The MME predictive skill is substantially improved compared with the averaged skill for all individual models (Fig. 3a,b). However, the ranges of time-averaged ACCs between the observed and model simulated precipitation anomalies in the two monsoon regions for different seasons show that the MME prediction skill is not necessarily better than a single model (Fig. 3c,d). The figure indicates that the best model or an ensemble of a few models can outperform the MME during a specific season.

Also, the MME predictive skills are relatively bounded for some seasons and regions. The skills of individual models and their MME’s precipitation prediction for Australasia and East Asia show seasonal dependence, with low skill during the boreal spring to summer and high skill during winter. During the boreal winter, the interannual variation of the predictive seasonal ACC skill of MME hindcast clearly and significantly depends on the fluctuation of ENSO amplitude (red dashed lines in Fig. 3; see also Fig. 1). The high skill is a result of the models’ capacity in simulating the mature ENSO variability and robust atmospheric teleconnection to remote regions. The range of individual models’ precipitation prediction skills for both regions also shows the seasonal dependence, with large spread during the boreal spring to summer and small spread during winter.

**Figure 4:**
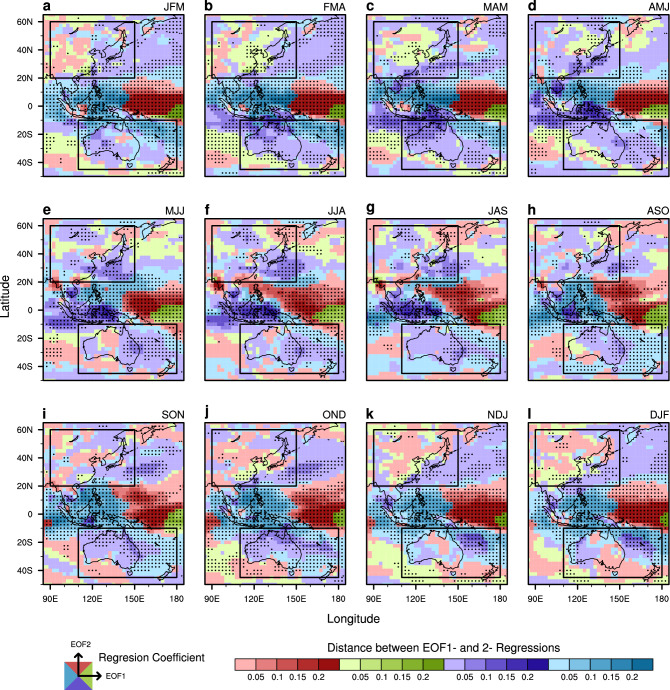
Relative signs and distance magnitudes (see the relevant coordinate and scale at bottom, respectively) on a 2-dimensional plane depicting regression coefficients of the seasonal mean anomalous precipitation onto the first two PCs for (**a**–**l**) JFM to DJF. In the coordinate at the bottom left corner, respective regression coefficients for the 1st and 2nd EOF are presented onto the x-axis and y-axis. Green (blue) area shows the grid point for which the relative magnitude of regression coefficient for EOF 1 is bigger than that for EOF 2, as well as the regression value for EOF 1 is positive (negative). Red (purple) area shows the same as above-mentioned, but for EOF 2. The black dots indicated grid points for which any regression coefficient for EOF 1 and 2 exceeds the 5% significance level based on the two-tailed Student’s *t* test. Two study domains, *i.e.* Australasia and East Asia, are denoted by the rectangle surrounded by black solid lines. The figure was generated by NCL.

Motivated by the above examination, an optimal combination of models is configured to enhance the MME seasonal precipitation prediction skill over the two regions. The individual models’ reproducibility of association between ENSO and precipitation variability is used as criteria for model selection. Also, ENSO is defined (Data and evaluation methods) to include the interannual change of the magnitude and position of seasonal mean SSTAs in the tropical Pacific, which induces the associated atmospheric response^19^. Figure 4 shows the observed precipitation anomalies associated with the dominant two modes of ENSO in the Asian-Australasian monsoon region, on a 2-dimensional plane depicting regression maps of precipitation onto the first two leading PCs. Green (blue) area shows the grid point for which the relative magnitude of regression coefficient for the first leading mode is bigger than that for the second mode, as well as the regression value for the first mode is positive (negative). Red (purple) area shows the same as above-mentioned, but for the second mode. The response of precipitation to ENSO exhibits large spatial variation even during the mature ENSO season. For instance, during the JFM season, anomalous rainfall surplus and deficit over East Asia and Australasia are more associated with zonal shift of the Pacific SST signal (*i.e.*, second mode) rather than the warming or cooling of the typical ENSO (*i.e.*, first mode). On the other hand, rainfall change in the adjacent ocean area to Australia is more related with warming or cooling signals over the central to eastern Pacific. The response of precipitation to ENSO also demonstrates strong temporal variation. For example, by comparing JFM and FMA seasons, the impact of the central-to-eastern Pacific SST warming on the East Asian precipitation becomes larger than the impact of zonal shift. Furthermore, when comparing JFM with AMJ, the Australasian precipitation anomalies during boreal winter (spring) are more related to signals (zonal shift) of the central-to-eastern Pacific SSTs.

**Figure 5:**
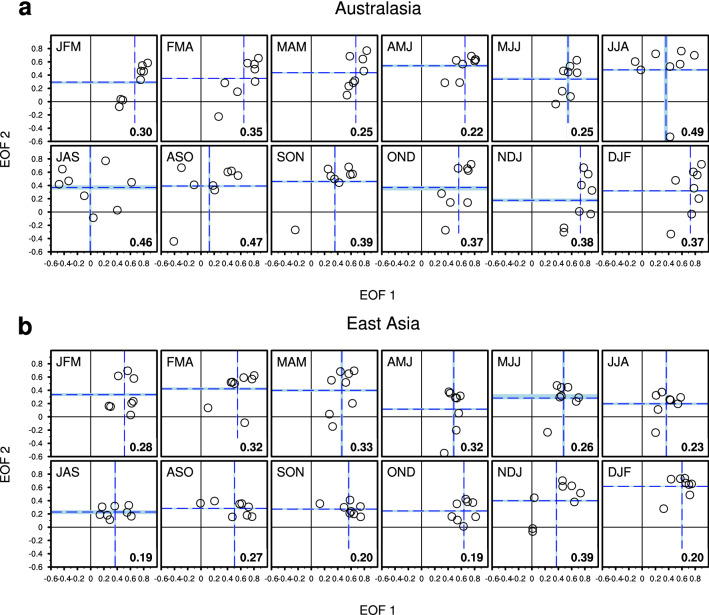
Scatter plots of time-averaged ACC skills (circles) between observed and model simulated regression patterns of (**a**) Australasian and (**b**) East Asian precipitation onto the respective first (x-axis) and second (y-axis) PCs of the ENSO. Individual panels of (**a**, **b**) denote the 12 calendar seasons from JFM to DJF. Blue dashed lines indicate the average skills of eight models used. The area filled in light blue indicates the ACC skill distribution of cross-validated hindcasts associated with the targeted season and year of prediction. The “standard distance deviation” of both EOF modes is given at bottom right of each panel. The figure was generated by NCL.

To examine models’ capability in capturing the spatial associations with the two leading modes of ENSO, ACCs between the observed and simulated regression maps are calculated (shown in Fig. 5). In general, the standard distance deviation of both EOF modes during boreal summer is larger than that during winter. For East Asia, the standard distance deviation is larger during boreal spring. For the JFM season, precipitation patterns over Australasia associated with the warming (or cooling) in the central-to-eastern Pacific from each model well capture that of observations. However, it seems relatively difficult to reproduce the impact of zonal shift associated with the second mode onto Australasian precipitation in the model simulation. On the other hand, for the JJA season, the spread of fidelity among models for the first mode becomes larger, and the ability of the model in reproducing the precipitation pattern related to the second mode becomes slightly better. Due to the different model performance on temporal and spatial association between ENSO and precipitation, the models exceeding the averaged skills are selected to configure an optimal MME.

### Better prediction of regional rainfall using seasonal dependence

**Figure 6:**
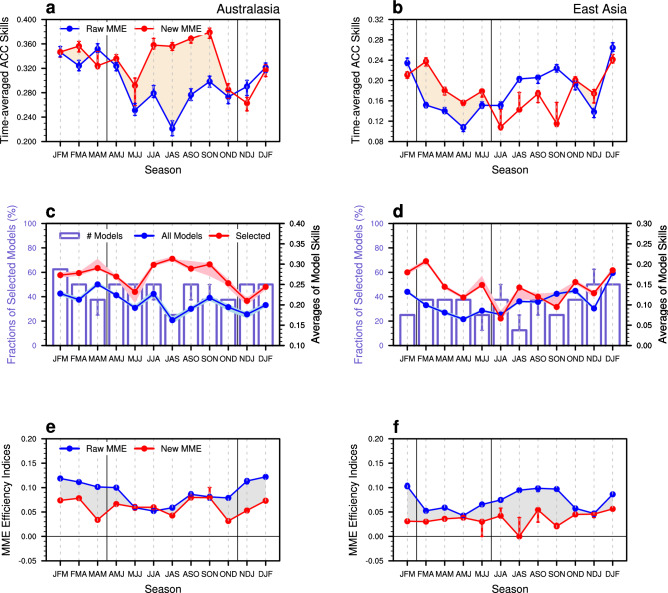
(**a**, **b**) Time-averaged ACC skills of the two MME mean (**a**) Australasian and (**b**) East Asian precipitation predictions as a function of seasons. Blue and red circled lines denote the raw MME based on all-inclusive models and new MME based on best performing models, respectively. Peach area indicates the improved prediction skill of new MME from raw MME mean. (**c**, **d**) Same as (**a**, **b**), but for fractions of selected model for the new MME mean (units: %; bar for left y-axis) and the averaged skills (right y-axis) of all models (blue circle line) and selected models (red circled line). (**e**, **f**) Same as (**a**, **b**), but for MME efficiency indices (see the text for details). Grey area indicates the decreased value for MME efficiency indices of new MME from raw MME mean. The vertical solid lines in black present the reference periods (**a**, **c**, **e**) from AMJ to OND for Australasian and (**b**, **d**, **f**) from FMA to MJJ for East Asian precipitation predictions for comparison. The vertical ranges in blue and red indicate the distribution of cross-validated hindcasts associated with the targeted season and year of prediction, for (**a**–**d**) the ACC skills and (**e**, **f**) MME efficiency indices. The figure was generated by NCL.

The optimization of MME through model selection improves the cross-validated predictive skills at regional levels, compared to those for all-inclusive MME, especially during boreal spring to summer (Fig. 6). The core predictability source in DSP, ENSO, dominates during the boreal winter. However, it is mostly during the non-mature phase of ENSO seasons that the better prediction of ENSO complexity and its climate impact lead to better prediction of regional precipitation variability (in particular, in Australasia for AMJ to OND and in East Asia for FMA to MJJ). Stationary skill improvements of the new MME during these seasons are found by simply partitioning a sample of data into training and test set (see Supplementary Figure S1). When configuring the sub-set of seasonal climate prediction models with the best representation of the association of precipitation changes with ENSO, 25 ~ 50% and 25 ~ 40% of the models are selected for Australasia and East Asia, respectively. At the expense of the number of models used, the average skills of individual models increase during the non-mature phase of ENSO seasons. Therefore, the MME efficiency, defined by the difference between the MME’s skill and averaged skill of individual models, is not much different between before and after the model selection during the non-mature phase of ENSO seasons.

## Conclusions

The Australasian and East Asian precipitation changes associated with the ENSO variability are examined, which is used to evaluate the regional predictability of general circulation models participating in the APCC MME seasonal climate prediction during 1982–2005. Specifically the individual models are assessed and selected based on the Australasian and East Asian precipitation variation in response to the magnitude and position of tropical SSTAs, which is identified by the first two leading EOF modes of the tropical Pacific SSTAs, respectively.

The prediction skill of the new combination of the models with best representation is much higher than that of the original MME with all available models in predicting the Australasian and East Asian precipitation variability during the non-mature phase of ENSO seasons. Although the leading mode that is characterized by central-to-eastern equatorial SSTA is relatively well represented by the models especially during boreal winter (Fig. 2), the ENSO impact on regional precipitation over Australasia and East Asia is highly modulated by the second mode that is characterized by the zonal shift of SSTA (Fig. 4). The second mode and the associated precipitation response dominate the non-mature seasons of ENSO (Fig. 2), but the model spread is large (Fig. 3c,d) during these seasons. By configuring MME based on the models’ performance (Fig. 5), the optimal MME shows significantly improved predictive skill during the non-mature seasons of ENSO (Fig. 6). The MME efficiency shows that the improvement originates from the average improvement of participating models’ performance, especially for the non-mature season of ENSO when the second mode is active.

The performance of the MME seasonal climate prediction depends on the number of models being composited^20^, individual models’ performance and the mutual independence^21^. The mutual association of predictive skills of individual models does not necessarily contribute more to the predictive skill in the MME (Supplementary Figure S2). Since the MME method has been developed during the late 1990s, the controversial issue has been still focused on whether the MME skill improves as the number of models being composed increases and whether the MME skill varies with different combinations of the model being composed. This study explored how we get better performance in predicting the Australasian and East Asian precipitation variability during the non-mature phase of ENSO seasons with the optimal MME despite decreasing the number of models.

The ENSO defined in this study has seasonality in its presence and predictability. There is large variance (high prediction skill) for boreal winter and small variance (low skill) for spring and summer for the first mode, and the opposite for the second mode. The regional precipitation prediction skills for Australasia and East Asia also show such seasonality, with low skill (large spread of individual models’ skills) during the boreal spring to summer and high skill (small spread) during winter. The increased prediction skill of the optimal MME relative to the original MME during the non-mature phase of ENSO seasons is related to the combination of seasonal features between ENSO variability and regional precipitation variation as well as its reproducibility of individual models. During the non-mature phase of ENSO seasons, the first and the second modes have smaller and larger contributions to the predictability, respectively, and consequently the systematic bias becomes the larger in DSP. Therefore, the key factor to improve the seasonal climate prediction would be to realistically reproduce the dominant features of ENSO events and the climate impact.

This study is subject to the limitation of hindcast data length, and assumption of the stationarity of ENSO. However, the ENSO prediction skill has been reported to decrease during the recent decade (2002–2011)^22^ due to reduced ENSO amplitude and more frequent occurrence of different types of ENSO events. Therefore, it needs further insight on how the model selection can be generalized to retrospective forecasts for different reference periods or independent real-time forecasts. In addition, ENSO is not the only driver for regional climate. Other climate drivers and external forcings, such as interactive sea-ice coupling and realistic land surface initialization^23^, should be thus continuously considered in DSP. The impact on prediction skill of regional climate should also be accessed in a MME framework, to get better seasonal predictive accuracy on regional level.

### Data and evaluation methods

The ENSO events and its associated precipitation impact over the two monsoon regions are diagnosed based on the observed datasets^24,25^ and seven models of APCC MME seasonal climate prediction (Supplementary Table S1), for the period covering 1982–2005. To implement the fair evaluation on climate models’ performances and doable MME calculation, a common period among models and observations is selected as a reference. Observed global SSTs are the improved analysis based on in situ and satellite data using the optimum interpolation^24^. The observed rainfall product is the Climate Anomaly Monitoring System (CAMS) and Outgoing Longwave Radiation (OLR) Precipitation Index (OPI) (CAMS OPI), created based on data from rain gauge and estimates from satellites^25^. Hindcast datasets from seven different tier-1 models are analysed based on one-month lead monthly rolling three-month means from JFM to DJF seasons. All observational and model datasets are analysed at 2.5° spatial resolution.

The EOF analysis of the seasonal mean SSTAs over the tropical Pacific Ocean (25°S–25°N, 140°E-80°W) on a monthly basis is carried out to define the state of ENSO. The ENSO in the present study is defined by superposition of the first two EOF modes^14^ (Supplementary Figure S3 and Figure S4) to capture a mixture of eastern Pacific and central Pacific ENSO. The first spatial structure is characterized by the warming (cooling) in the central-to-eastern tropical Pacific. The second mode is orthogonal to the first mode, indicating the zonal shift. The larger positive (negative) phase indicates the more westward (eastward) shift of the warming in the central-to-eastern tropical Pacific.

The reproducibility of association between two modes of ENSO and the regional rainfall is used to evaluate the models’ performance and to select skilful models for new MME subset. To ensure the statistical stationarity of raw and new MME, as well as to avoid the artificial skill, over-fitted association, and arbitrary model selection, the standard leave-one-out cross-validation method^26,27^ is applied throughout the work including anomaly and EOF calculation, evaluation of reproducibility, and verification of predication skill for both retrospective forecast and observations. For instance, the seasonal mean anomaly for one targeted year is computed from the corresponding climatological seasonal mean during the rest reference period excluding the targeted year. We found that the prediction skill of targeted season and year is within the range of cross-validated hindcast skill, and the difference from the cross-validation is in general trivial. To quantify the 2-dimensional dispersion of a distribution of models’ performance in reproducing the association of regional rainfall with both EOF modes, the “standard distance deviation” in a coordinate plane of pattern correlation for the 1^st^ (x axis) and 2^nd^ (y axis) modes is calculated. It is given by

$$\sqrt {\frac{{\mathop \sum \nolimits_{i = 1}^{n} \left( {x_{i} - \overline{x}} \right)^{2} + \mathop \sum \nolimits_{i = 1}^{n} \left( {y_{i} - \overline{y}} \right)^{2} }}{{\text{n}}}},$$

and it represents the standard deviation of the distance of each point from the mean centre.

## Supplementary information

Supplementary Information 1.
