# Supplementary material for: Toward a better multi-model ensemble prediction of East Asian and Australasian precipitation during non-mature ENSO seasons
Source: Sci Rep. 2020 Nov 20;10:20289. doi: 10.1038/s41598-020-77482-4 (PMC7679460; doi:10.1038/s41598-020-77482-4)
Supplement: Supplementary file 1 — Supplementary Information 1. [file 41598_2020_77482_MOESM1_ESM.docx]

Supplementary Material for

**Toward a better multi-model ensemble prediction of East Asian and Australasian precipitation during non-mature ENSO seasons**

**Soo-Jin Sohn^*^and WonMoo Kim**

Prediction Research Department, Climate Services and Research Division, APEC Climate Center (APCC), Busan, Republic of Korea

Contents of this file

References (cited in *Supplementary Material*)

Figures S1- S4

Table S1

Introduction

*Supplementary Material* (e.g. Figures and Table) provides auxiliary information cited in the main article.

References

1. Cottrill, A. *et al.* Seasonal forecasting in the Pacific using the coupled model POAMA-2 *Weather and Forecast.* **28**, 668-680 (2013).
2. Merryfield, W. J. *et al.* The Canadian seasonal to interannual prediction system. Part I: Models and initialization. *Mon. Wea. Rev.* **141**, 2910-2945 (2013).
3. Alessandri, A. *et al.* The INGV-CMCC Seasonal Prediction System: Improved ocean initial conditions. *Mon. Wea. Rev.* **138**, 2930-2952 (2010).
4. Takaya, Y. *et al.* Japan Meteorological Agency/Meteorological Research Institute-Coupled Prediction System version 2 (JMA/MRI-CPS2): atmosphere-land-ocean-sea ice coupled prediction system for operational seasonal forecasting. *Clim. Dyn.* **50**, 751-765 (2018).
5. Ham, S., Lim, A.-Y., Kang, S., Jeong, H. & Jeong, Y. A newly developed APCC SCoPS and its prediction of East Asia seasonal climate variability. *Clim. Dyn.* **52**, 6391-6410 (2019).
6. Ham, Y. G., Schubert, S. & Chang, Y. Optimal initial perturbations for ensemble prediction of the Madden-Julian oscillation during boreal winter. *J. Clim.* **25**, 4932-4945 (2012).
7. Ham, Y. G., Schubert, S., Virkhliaev, Y. & Suarrez, M. J. An assessment of the ENSO forecast skill of GEOS-5 system. *Clim. Dyn.* **43**, 2415-2430 (2014).
8. Saha, S*. et al.* The NCEP Climate Forecast Version 2. *J. Clim.* **27**, 2185-2208 (2014).

**Table S1.** Description of the seven coupled climate models used in this study.

| Country | Institute | Model | AGCM/resolution | OGCM/resolution | Ensemble Member | Hindcast Period |
| --- | --- | --- | --- | --- | --- | --- |
| Australia | BOM | POAMA2.4**^1^** | BAMv3.0d/T47L17 | ACOM2/0.5-1.5°lat x 2°lon L25 | 33 | 1983-2011 |
| Canada | MSC | CCCma CGCM**^2^** | AGCM3/T63L31 | OGCM4  (1.41°lon x 0.94°lat L40) | 10 | 1982-2010 |
|  |  |  | AGCM4/T63L31 |  | 10 | 1982-2010 |
| Italy | CMCC | CMCC-SPSv2**^3^** | ECHAM5.3/T63L19 | OPA8.2/ORCA2 grid_L31 | 9 | 1982-2005 |
| Japan | JMA | JMA/MRI-CPS2**^4^** | JMA-GSM/TL159L60 | MRI.COMv3/0.3-0.5x1.0°L53 | 10 | 1979-2014 |
| South Korea | APCC | SCoPS**^5^** | ECHAM5.3/T159L31 | POP2.0.1/0.3-0.5x1.0°L40 | 5 | 1982-2013 |
| USA | NASA | GMAO**^6-7^** | GEOS-5/288x181L72 | MOM4/720 x 410 L40 | 11 | 1982-2011 |
|  | NCEP | CFSv2**^8^** | GFS/T126L64 | MOM4/⅓°lat x 1°lon L40 | 20 | 1982-2010 |

**
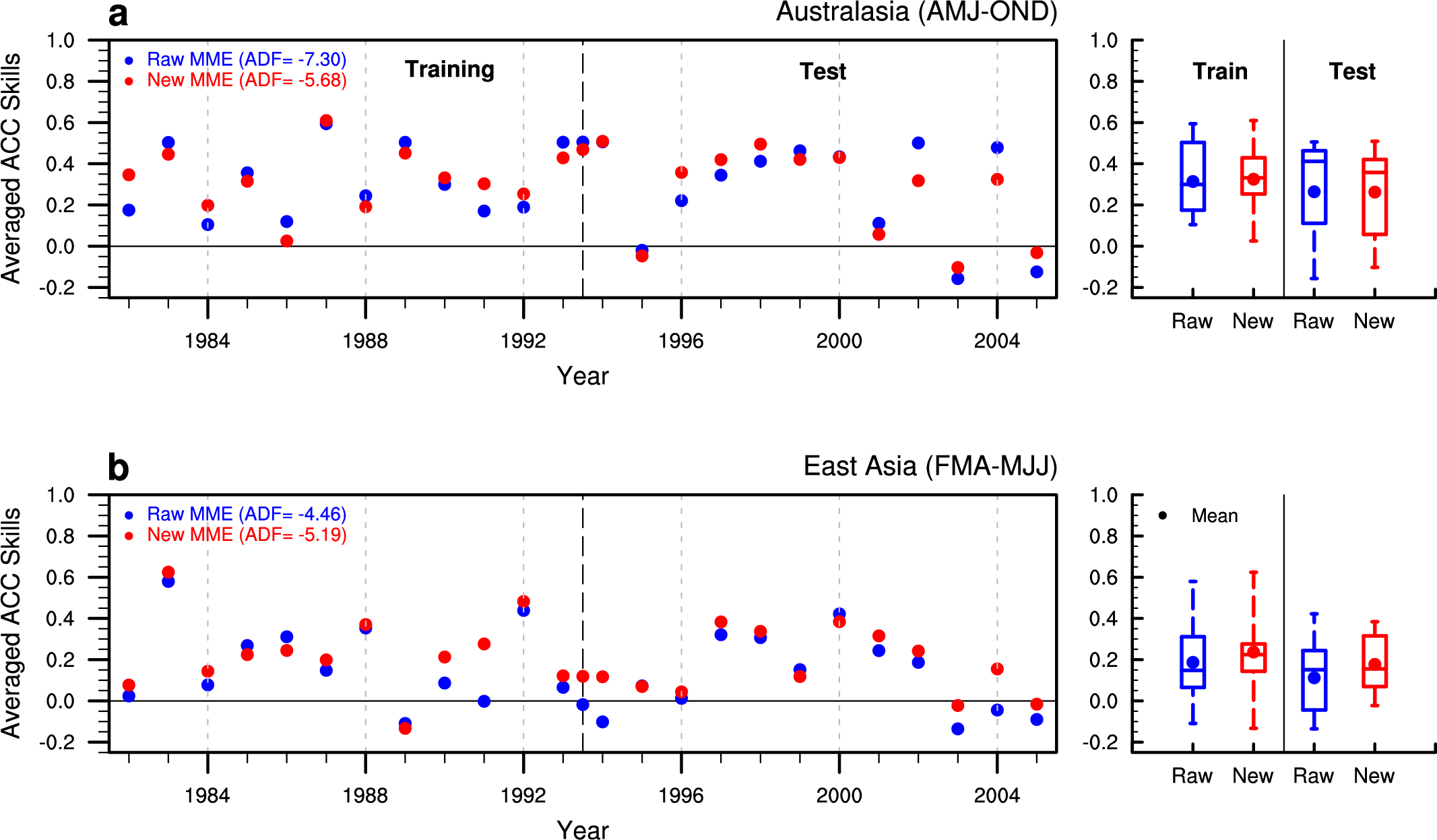
**

**Figure S1.** Time series (left panels) and their statistical distribution (right panels) of interannual variation of ACC skills averaged during corresponding AMJ-OND and FMA-MJJ seasons to (a) Australasian and East Asian precipitation predictions, based on training (1982-1993) and test (1994-2005) periods. Blue and red circles denote the raw MME based on all-inclusive models and new MME based on best performing models, respectively. Augmented Dickey-Fuller (ADF) tests are applied to test the stationarity of whole time series, and the relevant ADF statistics are provided in parentheses following the labelled targeted data sets of upper left of each panel. The statistical distribution of these time series for the raw and new MMEs are illustrated by box plots and mean values (circles), based on training and test periods.

**
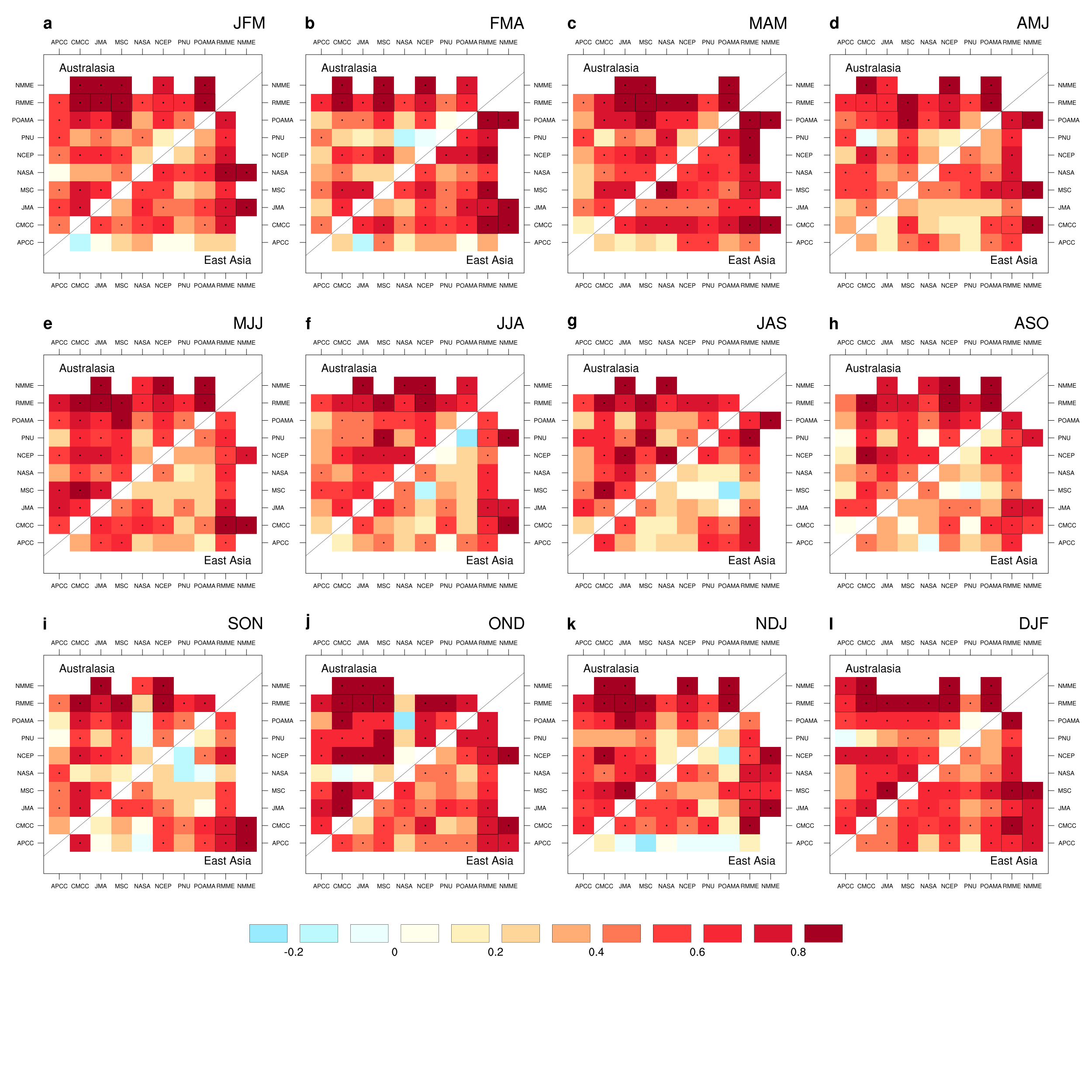
**

**Figure S2.** Mutual correlation coefficients (shading; see scale at bottom) between individual models and MMEs for time series of ACC skills of on Australasian (above the diagonal line) and East Asian (below the diagonal line) precipitation prediction for (a-l) JFM to DJF during the period of 1982 to 2005. The black dots indicated points for which any correlation coefficient exceeds the 95% significance level based on the two-tailed Student’s *t* test. Acronyms of “RMME” and “NMME” on the both of x-axis and y-axis denote the raw MME based on all-inclusive models and new MME based on best performing models, respectively. The selected models are denoted by the squares on RMME row and column. The figure was generated by NCL.


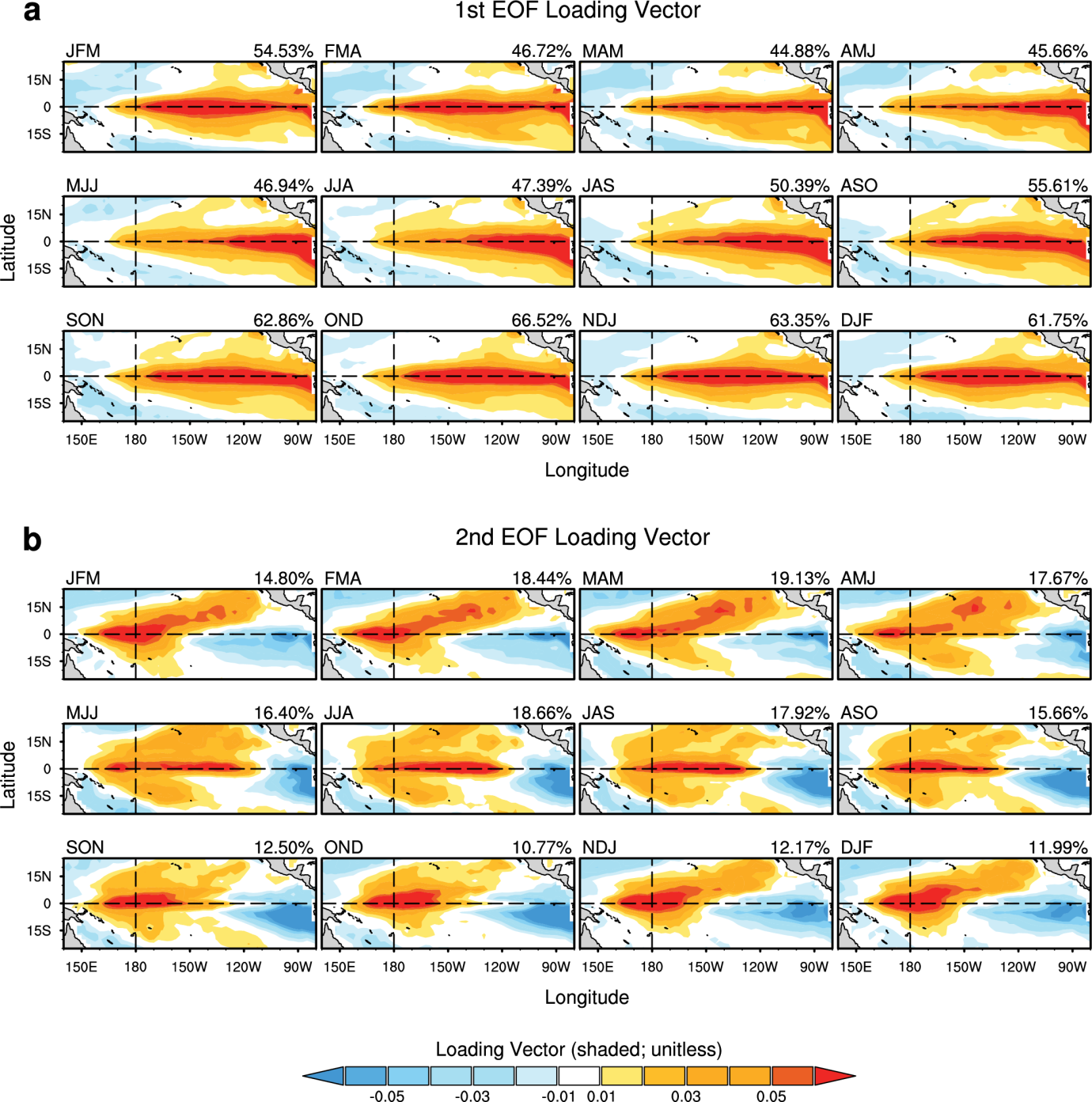


**Figure S3.** (a) First and (b) second EOF patterns of a monthly rolling 3-month mean SSTAs computed for 25°S–25°N and 140°E–80°W during 1982–2005, based on OISST. The fractional variance explained by each mode and season are provided in the upper right of each panel. The horizontal and vertical black dashed lines denote the equator and International Date Line. The figure was generated by NCL.


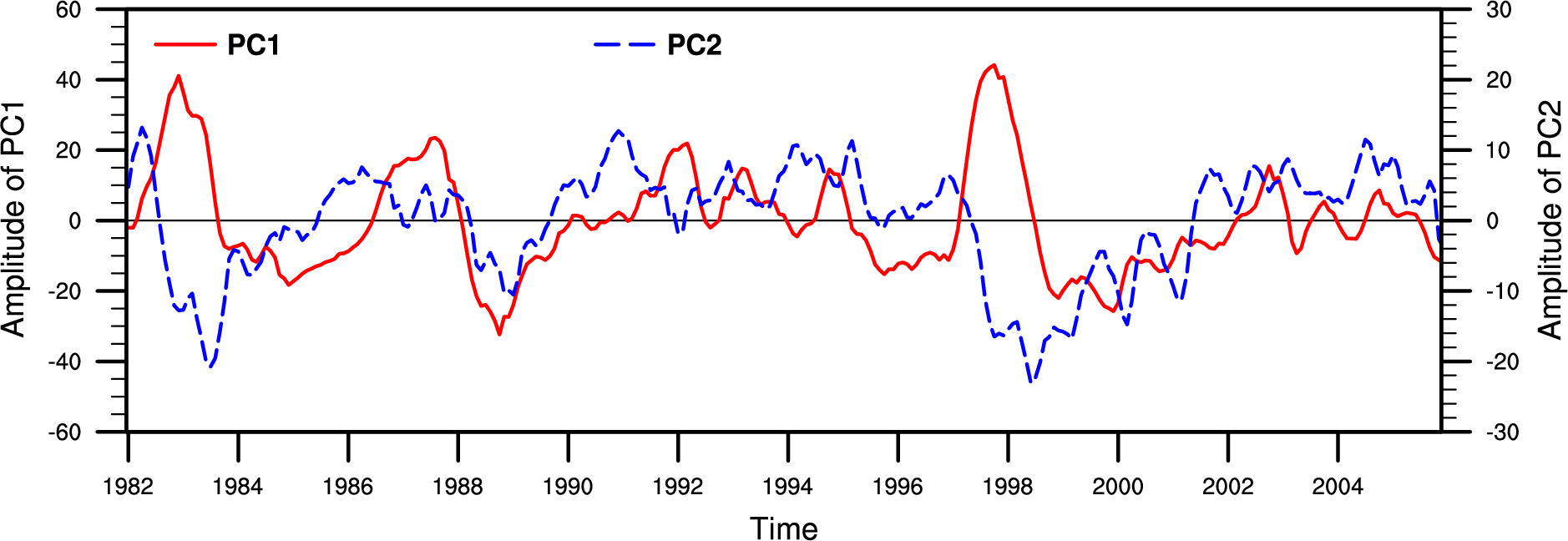


**Figure S4**. Time series of the combined PCs of (a) the first mode (red solid line for left y-axis) and (b) the second mode (blue dashed line for right y-axis) associated with **Figure S3** with all months during January 1982 to December 2005. The figure was generated by NCL.
